# Extending the resolution limits of nanoshape imprint lithography using molecular dynamics of polymer crosslinking

**DOI:** 10.1038/s41378-020-00225-y

**Published:** 2021-02-01

**Authors:** Anshuman Cherala, Parth N. Pandya, Kenneth M. Liechti, S. V. Sreenivasan

**Affiliations:** NASCENT Engineering Research Center, The University of Texas at Austin, Austin, TX USA

**Keywords:** Nanoscale devices, Nanoscale materials

## Abstract

Emerging nanoscale applications in energy, electronics, optics, and medicine can exhibit enhanced performance by incorporating nanoshaped structures (nanoshape structures here are defined as shapes enabled by sharp corners with radius of curvature < 5 nm). Nanoshaped fabrication at high-throughput is well beyond the capabilities of advanced optical lithography. Although the highest-resolution e-beams and large-area e-beams have a resolution limit of 5 and 18 nm half-pitch lines or 20 nm half-pitch holes, respectively, their low throughput necessitates finding other fabrication techniques. By using nanoimprint lithography followed by metal-assisted chemical etching, diamond-like nanoshapes with ~3 nm radius corners and 100 nm half-pitch over large areas have been previously demonstrated to improve the nanowire capacitor performance (by ~90%). In future dynamic random-access memory (DRAM) nodes (with DRAM being an exemplar CMOS application), the implementation of nanowire capacitors scaled to <15 nm half-pitch is required. To scale nanoshape imprint lithography down to these half-pitch values, the previously established atomistic simulation framework indicates that the current imprint resist materials are unable to retain the nanoshape structures needed for DRAM capacitors. In this study, the previous simulation framework is extended to study improved shape retention by varying the resist formulations and by introducing novel bridge structures in nanoshape imprinting. This simulation study has demonstrated viable approaches to sub-10 nm nanoshaped imprinting with good shape retention, which are matched by experimental data.

## Introduction

Applications in the areas of energy storage^1,2^, nanoscale photonics^3^, plasmonic structures^4^, multibit magnetic memory^5^, terabit per sq. in. magnetic recording^6^, and bionanoparticles^7,8^ require high-throughput patterning and complex shape control at the nanoscale.

Among nanofabrication techniques, the state-of-the-art form of optical lithography—193 nm immersion (193i) lithography—has plateaued at a resolution of ~38 nm half-pitch for gratings and ~50 nm half-pitch for more complex structures. Higher-resolution large-area patterns are currently manufactured by complementing photolithography (PL) with self-aligned double patterning (SADP) and multiple lithography-etch steps. Directed self-assembly (DSA) is also being explored; however, both SADP and DSA are primarily restricted to periodic features^9–11^. Although the highest-resolution e-beam processes (Gaussian beam tools with nonchemically amplified resists) can achieve <5 nm resolution^12–14^, this is only available at very low throughputs. Large-area e-beam fabrication using variable shape beam tools, needed for photomasks and imprint templates, is limited to ~18 nm half-pitch lines and spaces^15^, and ~20 nm half-pitch hole patterns^16^. Other techniques such as extreme ultraviolet lithography and multiple e-beam lithography face fundamental challenges such as lack of light source, line edge roughness, and low throughput ^17,18^.

Imprint lithography has demonstrated large-area patterning at sub-10 nm half-pitch, with the capability to pattern typical lithographic structures including lines, gratings, dot arrays, etc^19–22^. Due to its near-molecular-level resolution over large areas and its progress in scalability, jet and flash imprint lithography (J-FIL) is a viable candidate for manufacturing sub-20 nm patterns in semiconductor devices^23^ and for sub-10 nm patterns in hard disks^24^. In J-FIL, a low-viscosity resist is deposited onto the substrate using an inkjet dispenser. This dispensing technique has been chosen in J-FIL to match the distribution of resist to the pattern density variation in the template, which enables high-throughput patterning of arbitrary structures. The patterned template is then lowered onto the dispensed material on the substrate so that the relief patterns are filled by capillary action. The resist material, which is an acrylate-based multicomponent formulation, is then crosslinked under ultraviolet (UV) radiation. Finally, the template is removed, leaving a patterned resist on the substrate. The exposure dose in UV-crosslinked nanoimprints (NILs) needs to be above a threshold, to ensure sufficient availability of photons needed for crosslinking^25^. This approach is therefore different from PL, where the dose of the exposure influences the critical dimension that results after the exposure^26^. In addition, in PL, the photoresist film is a spin-coated film, which requires the use of a developer to remove the exposed region of the photoresist (assuming that the photoresist is a positive resist). This removal of the exposed regions results in the relief structure that is subsequently used during the etch step. In NIL, there is no developer involved, as the patterning step creates a relief image that is a negative replica of the template (or mold). The specific questions addressed in this study are as follows: Assuming that the dose is above the required threshold, what is the extent of crosslinking that can be achieved in nanoshape polymeric structures? This crosslinking in the nanoshape structures is not limited by dose but by the availability of double-bonded carbon atoms in the vicinity of the crosslinker. For our material, ethylene glycol diacrylate is the crosslinker.

To demonstrate applications of nanoshape structures in energy storage, nanowire capacitors with diamond-like cross-sections and a half-pitch of 100 nm have been previously fabricated over large areas with the help of J-FIL and metal-assisted chemical etching, producing a 90% increase in performance compared to that of conventional circular cross-section nanowire capacitors^1^. Scaling the half-pitch down to 10 nm is expected to increase the performance by 10× and is needed for applications such as DRAM. However, the phenomenon of resist polymer relaxation has led to a limit in shape retention at these length scales^27,28^. This limitation necessitates developing a sophisticated model to capture shape retention and an all-atomistic model was developed to study crosslinked polymer relaxation. All-atom molecular dynamics (MD) models of nanoshape resist have been built in LAMMPS^29–33^ and validated by simulating the tensile test and glass transition temperature, and comparing with the literature^34^. Upon simulation of resist relaxation after UV crosslinking and template separation, the resist properties at the nanoscale were identified as a limitation for shape retention. Therefore, various techniques for improving shape retention in nanostructures, such as resist tone inversion, adding subresolution features and etch compensation, have been previously identified.

In this work, the atomistic model developed can be further enhanced to study shape retention as a function of resist formulation. The following sections expand on previous work exploring nanoshape retention in the sub-10 nm regime using MD simulations, with a focus on the extent of crosslinking in nanoshapes. In the section “Diamond-like nanoshape for the 13 nm half-pitch DRAM node”, the extent of crosslinking in a nanoshape structure as a function of various parameters, such as feature geometry, feature size, and distance from edge, is presented and alternate resist formulations to improve shape retention are proposed. Section “Crosslinking in nanoshaped structures” demonstrates improved crosslinking and shape retention in the nanoshape structures connected to each other using a sacrificial bridge structure. Section “Improving shape retention using sacrificial structures” discusses the effect of residual layer thickness (RLT) on shape retention.

## Diamond-like nanoshape for the 13 nm half-pitch DRAM node

**Fig. 1:**
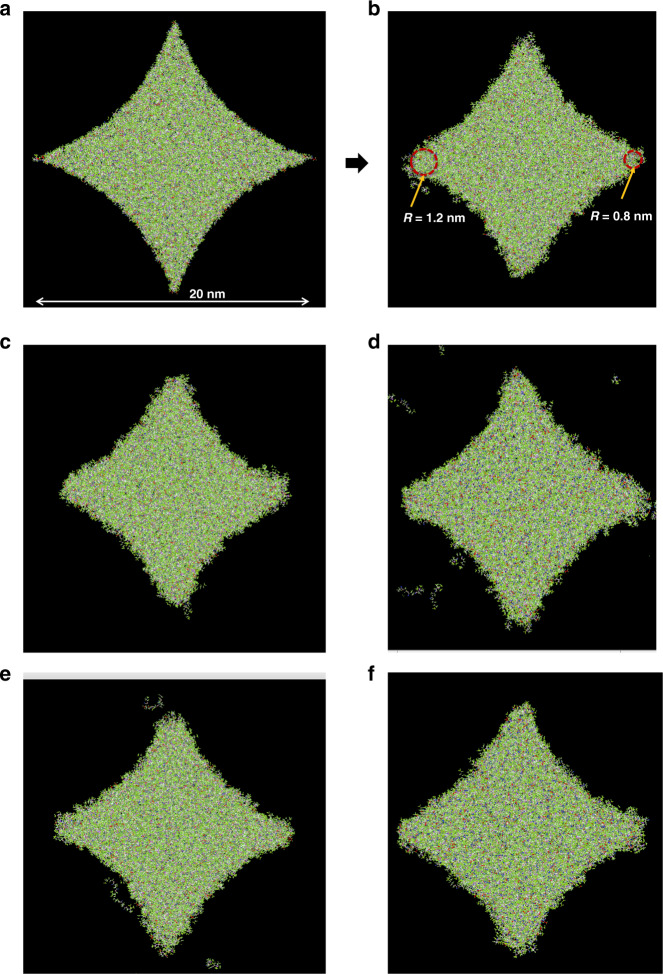
MD ensembles. **a** Example 20 nm diamond initial configuration. **b**–**f** Five ensembles after 50 ps simulation.

In the course of investigating nanoshaped structures, the statistical nature of observables becomes evident. For example, the corner radii of the nanoshaped diamond structures in the previous section are not uniform. The variation in radii comes from local non-uniformities in molecular spatial arrangement, bonding efficiencies, and local position and velocity (temperature) distributions. Therefore, to estimate the corner behavior characteristic of the “average” structure, several different structures, each with unique molecular arrangements, can be prepared and simulated. The average radius of the corner can then be calculated from different arrangements. To study this approach, five unique 20 nm diamond structures were prepared and simulated to 50 ps. These 20 nm diamond shapes approximately represent a 13 nm half-pitch DRAM deep trench capacitor design^2^. Figure 1 shows an exemplar initial diamond structure in inset (a) and the five simulated structures (insets b–f). The average radius is estimated to be ~1.2 nm with an SD of ~0.2 nm. Statistical analysis can be conducted on this data set, to evaluate whether the differences in the mean radii at the four corners are statistically significant (the null hypothesis). To that end, a paired *t*-test evaluation is conducted, comparing radii at corner 1 to corners 2, 3, and 4 in a pairwise manner. Table 1 shows the raw data, mean, SD, and *p*-value results from the analysis. As the three calculated *p*-values are all above the significance level of 0.05, it can be concluded that the difference in the mean radii of the four corners is not statistically significant.

**Table 1:** Paired *t*-test for comparison of the mean radius of four corners in a 20 nm diamond structure.

	Corner 1, nm	Corner 2, nm	Corner 3, nm	Corner 4, nm
Ensemble 1	1.2	0.8	1.1	1.6
Ensemble 2	1.2	1.4	1.1	1.4
Ensemble 3	1.5	1.1	1.4	1.4
Ensemble 4	1.2	1.2	1.2	1.1
Ensemble 5	1.2	1	1.4	1.4
Mean	1.3	1.1	1.2	1.4
SD	0.1	0.2	0.2	0.2
*P*-value, two-tailed (significance level = 0.05)		**0.24**	**0.75**	**0.28**

## Crosslinking in nanoshaped structures

A key parameter that is expected to influence the material modulus and strength in the resist is the crosslinking quality and uniformity across the nanoshape. As shown in Table 2, the percentage crosslinking of bulk (baseline) resist is 87%, and two nanoshapes, namely, 7.5 nm diamond and 10 nm cross-shapes, have crosslinking percentages of 75% and 60%, respectively. The shape and size dependence is evident in these data.

**Table 2:** Crosslinking percentage as a function of nanoshape.

MD may be used to estimate the quality and uniformity of crosslinking as a function of the shape and size of the feature. Although reverse tone and/or design for nanoshape retention (DNR) have been proposed as a potential solution to shape retention challenges, resist formulation can also be optimized for shape retention based on this investigation if tone inversion is not desirable^34^. It is hypothesized that the crosslinking of the nanoshape is worse than that of the bulk due to the shape and size influence. Crosslinking is a spatial phenomenon and the probability of bond formation at a point in the nanoshape is a function of the amount of material surrounding the point within a certain radius. In bulk resist, this probability is constant except for local stochastic variations. On the other hand, in a nanoshape, this probability is a function of both size and shape.

This phenomenon was studied by performing crosslinking studies (using the MD framework) of diamond and cross nanoshapes with various sizes starting from 25 nm and decreasing to 5 nm, and calculating the crosslinking percentage as a function of size and shape. The crosslinking percentage is calculated based on the number of carbon atoms with newly formed single bonds after crosslinking with respect to the initial number of double-bonded carbon atoms in the monomer model^34^.

**Fig. 2:**
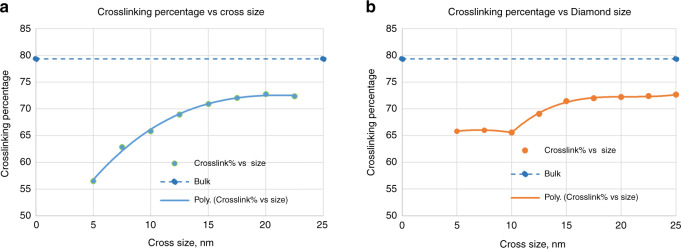
Crosslinking percentage as a function of size. **a** Cross-nanoshape and **b** diamond nanoshape.

Figure 2 below shows the results after simulation and data analysis.

The crosslinking quality degrades with reducing nanoshape size and does not reach the bulk crosslinking value (79%), even for the largest nanoshape sizes. Further, the cross and diamond crosslinking curves are not identical, especially below 10 nm. This observation clearly shows that nanoshape size and shape adversely affect bonding below 25 nm. It is believed that the difference in the crosslinking percentage curves between the cross and diamond nanoshape is primarily driven by the shape differences, which in turn influence the average number of carbon double-bond neighbors available for crosslinking. Furthermore, the diamond crosslinking curve plateaus at a crosslinking percentage at 65% below the 10 nm critical dimension. It should be noted that the bulk crosslinking value reported here (79%) is different from the value reported earlier (~87%). This difference is due to a decision made to limit the bonding time for computational cost reasons.

### Bonding efficiency as a function of size of the nanoshape structure

**Fig. 3:**
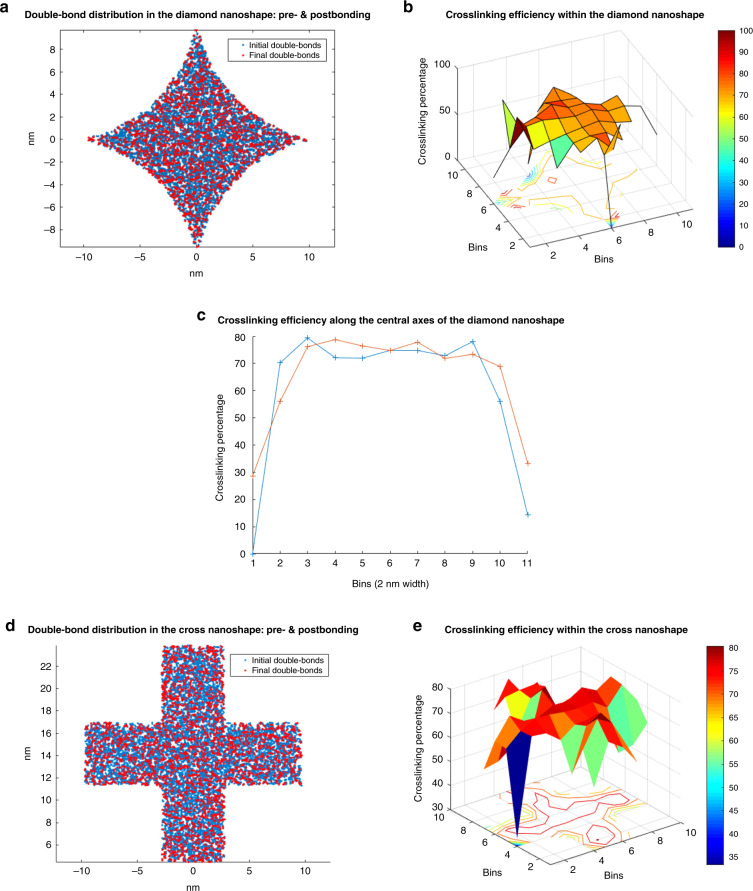
Spatial distribution of crosslinking efficiency. **a** The double-bonded carbon atom distribution in the diamond nanoshape before and after bonding, **b** the crosslinking efficiency within the diamond nanoshape, **c** along the central axes, **d** the double-bonded carbon atom distribution in the cross-nanoshape before and after bonding, and **e** the crosslinking efficiency within the cross nanoshape.

The spatial distribution of the uncrosslinked double bonds was studied for the 25 nm diamond and cross. The initial and final carbon double-bond locations are shown in Fig. 3a, b, respectively. The bonds are binned in 20 Å square bins before and after crosslinking (two-dimensional histogram in the *X*–*Y* plane). The crosslinking percentage can then be calculated for each bin and gives the spatial distribution of crosslinking. Figure 3d, e show the local crosslinking efficiency.

As seen, there is a strong dependence of crosslinking on the location within the nanoshape. In the diamond shape, the extent of crosslinking is similar to that of the bulk (~80%) near the center of the feature and sharply degrades at the corners (~30–60%).

In the diamond shape, the extent of crosslinking remains close to the bulk along the middle axes of the feature and drops off on either side of the axes towards the edge and corners.

This kind of crosslinking percentage information could be used to predict which shapes are difficult to achieve and require tone inversion or DNR techniques developed earlier.

### Computational design of resist for nanoshape structures

An important design parameter that has not been explored thus far is the composition of the imprint resist. In this section, the MD framework is used to inform the resist formulation itself.

The resist composition taken from the literature consists of three acrylate monomer molecules, namely hexyl acrylate (~55% w/w), isobornyl acrylate (~25% w/w), and ethylene glycol diacrylate (~20% w/w), as the crosslinker. The monomer mixture forms a low-viscosity (<10 cps) liquid comprising the above molecules, which are in the range of 0.5–1 nm in size. By virtue of the versatility of MD, it allows for the investigation of various resist material formulations purely in silico. It is proposed to leverage this computational material design capability to optimize the imprint resist formulation specifically for shape retention in nanoshapes.

More specifically, based on crosslinking studies conducted with imprint resist^35^, there is a correlation between the proportion of crosslinker in the resist and the crosslinking percentage. Higher crosslinker amounts lead to faster crosslinking but reduce the crosslinking percentage. These earlier studies were conducted on bulk resist materials. It is unknown how the crosslinker proportion impacts crosslinking in nanoshapes. It is proposed to use the MD design tool at our disposal to study the effect of crosslinker on crosslinking in cross and diamond shapes.

**Fig. 4:**
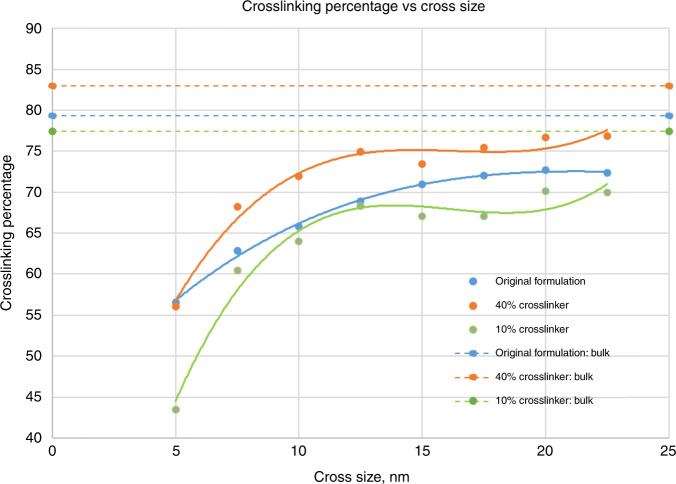
Resist Crosslinking in Nanoshapes. Crosslinking as a function of crosslinker and cross size.

For this study, the cross nanoshape size is chosen and two new resist formulations are created with 10% and 40% crosslinkers. Crosslinking is simulated with each new resist formulation to analyze the effect on the crosslinking percentage. Figure 4 summarizes the results of this analysis. The effect of formulation change on crosslinking roughly follows bulk resist behavior. For example, at the 10 nm dimension, the percentage increases from 66% to 72% with the new formulation. The increased crosslinker density helps with bonding efficiency by ~10%. This resist material design parameter for improving nanoshape retention has been examined here in an exploratory way. This exercise should be repeated for each new nanoshape design consideration.

It is noteworthy that all material properties such as modulus, strength, and *T*_g_ will have to be re-estimated for the new formulation(s), to ensure the performance in addition to increased crosslinking. In summary, the use of MD for computational material design has been demonstrated in this section. The addition of more crosslinkers enhances the crosslinking percentage; however, given that bulk crosslinking levels are not achieved by this method, it does not provide a complete solution for nanoshape retention.

## Improving shape retention using sacrificial structures

The previous section showed that bonding becomes progressively poorer near the edges and sharp features of nanoshapes. reactive-ion etching (RIE)-based DNR has been discussed earlier^34^ in the context of creating a sharp corner in 200 nm diamond fabrication. For smaller features, a similar idea can also be used, but in this case, with the help of the structural integrity of the nanoshape.

**Fig. 5:**
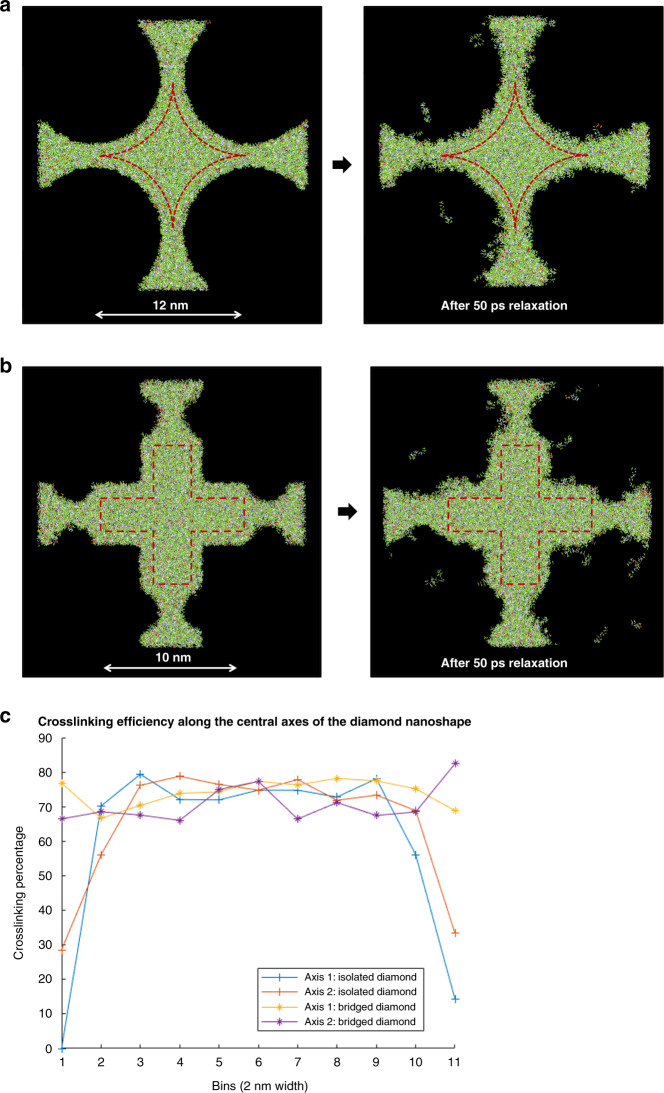
Using sacrificial bridge structures to overcome the bonding inefficiency. **a** Sacrificial bridge structures in the diamond nanoshape and **b** sacrificial bridge structures in the cross nanoshape. Crosslinking efficiency within the diamond nanoshape along the central axes is shown in **c**.

Figure 5a, b show a 10 nm diamond and cross nanoshape, respectively, with a 2 nm-wide bridge on all four sides, retaining its original geometry significantly better than an isolated nanoshape^34^. Figure 5c shows the bonding efficiency of an isolated diamond (same as in Fig. 3c) compared to that of a bridged diamond. The bridged diamond shows significantly better bonding across the entire central axis, thus confirming the structural integrity seen in the relaxation simulation. The bridge section of the feature may be removed after imprint replication by designing slightly isotropic etch chemistry. Such a slightly isotropic etch of a bridged nanoshape structure has been experimentally demonstrated in prior work^36^; see Fig. S1 in the Supplementary Information included with this paper. The target shape of the nanoshape after etching is shown by the red dotted lines. The feature on the other side of the bridge could be the neighboring nanoshape structure or a dummy feature (in case of a different pitch requirement).

## Effect of the RLT

**Fig. 6:**
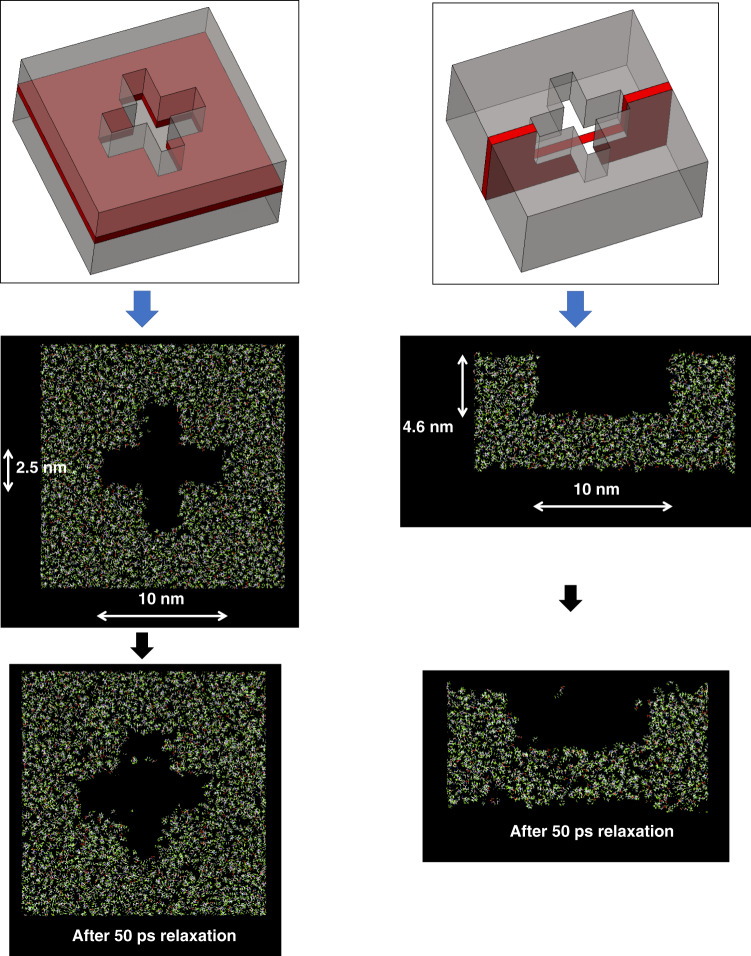
MD model of cross nanoshape with residual resist layer. Ten-nanometer-thick cross-section at the base of the cross hole nanoshape shown schematically in red (top) and the MD model before and after relaxation.

In all the previous simulations, an infinitely long feature (periodic boundary condition in the direction perpendicular to the plane of the figures) is assumed, to isolate the in-plane behaviors. The actual fabricated nanoshapes will, however, have a finite height and a RLT under the feature due to the nature of the J-FIL imprinting process. The effect of the RLT on nanoshapes is thus an important consideration. The cross nanoshape structure (10 × 2.5 nm) was simulated with an RLT in hole (reverse) tone and allowed to relax for 50 ps, as shown in Fig. 6. These figures show the resist behavior at the base of the cross hole nanoshape at a vertical cross-section through the middle of the feature. Nanoshape retention is generally similar to a cross hole with periodic boundary conditions.

## Conclusion

In a previous article, a nanowire capacitor with a diamond-shaped pattern possessing a 3 nm sharp corner radius and 100 nm half-pitch led to an improvement in the capacitance by 90% compared to the current state of the art, and a further 10× increase in performance was expected with 10 nm half-pitch patterns. However, exploring scaling down to a sub-10 nm half-pitch led to the discovery of a shape retention limit in the crosslinked polymer resist. In this article, improvements in the geometry of the patterned nanostructure using sacrificial structures and enhanced resist formulation have been identified to improve shape retention. Performing an MD study of crosslinking in nanoshapes as a function of size and shape has indicated that the extent of crosslinking decreases (w.r.t. bulk crosslinking) below a certain threshold size. The crosslinking percentage is significantly lower near the edges of nanoshapes. Simulations with a higher ratio of crosslinker have demonstrated greater crosslinking efficiency; however, this approach did not yield results that were comparable to the bulk crosslinking efficiency. On the other hand, the extent of crosslinking is shown to be greater in sacrificial bridges between the nanostructures and, in this case, is comparable to the bulk crosslinking percentage. Through the use of RIE to remove the bridges, shape retention can be improved due to the enhanced crosslinking percentage in the bridges. The RLT is found to have no significant impact on shape retention. In summary, this study provides insights into how nanoshape imprinting can be extended to sub-15 nm half-pitch structures.

## Supplementary information

Supplementary material for article titled, “Extending the Resolution Limits of Nanoshape Imprint Lithography Using Molecular Dynamics of Polymer Crosslinking”
