# Supplementary material for: Extending the resolution limits of nanoshape imprint lithography using molecular dynamics of polymer crosslinking
Source: Microsyst Nanoeng. 2021 Feb 1;7:13. doi: 10.1038/s41378-020-00225-y (PMC8433368; doi:10.1038/s41378-020-00225-y)
Supplement: Supplementary file 1 — Supplementary material for article titled, “Extending the Resolution Limits of Nanoshape Imprint Lithography Using Molecular Dynamics of Polymer Crosslinking” [file 41378_2020_225_MOESM1_ESM.docx]

**Supplementary material for article titled, “Extending the Resolution Limits of Nanoshape Imprint Lithography Using Molecular Dynamics of Polymer Crosslinking”**

Anshuman Cherala, Parth N. Pandya, Kenneth M. Liechti, S.V. Sreenivasan

NASCENT Engineering Research Center, The University of Texas at Austin

1. **Experimental data for etch of bridged structures in nanoshapes:**

Fig. S1 a) SEM image of diamond-like shaped template feature (left) with a 2.6 nm radius corner and corresponding feature after RIE etch (right) into silicon oxide with a 5.4 nm radius corner b) SEM image of diamond-like shaped template feature (left) with 3.0 nm bridge gap and corresponding feature after RIE etch (right) into silicon oxide with 4.5 nm radius corner^[[1]](#footnote-1)^

It is shown that the reactive ion etch (RIE) process that transfers the shape into the underlying substrate tends to have a small isotropic etch rate. This can be exploited by designing the template to have a thin connection between adjacent diamond features as shown in Figure S1 (b). This gap creates a bridge structure in the imprinted resist. The subsequent etching process breaks down the 3nm bridge leaving a sharper corner when compared to starting with an isolated diamond feature. Figure S1 a & b show an isolated diamond with 2.6 nm corner and diamond with a thin connecting bridge (left images) and the corresponding structures after etching into the underlying oxide layer (right images). As can be seen, the bridged diamond shows the sharper corner after RIE, due to the effect explained above.

1. Cherala, Anshuman. Nanoshape imprint lithography: fabrication and modeling. Diss. 2019. [↑](#footnote-ref-1)
